# Responsiveness and Relationship Satisfaction in Couples Coping With Parkinson’s Disease: A Pilot Study

**DOI:** 10.1177/0033294121998032

**Published:** 2021-02-24

**Authors:** Eden Rose Champagne, Amy Muise

**Affiliations:** Department of Psychology, York University, Ontario, Canada

**Keywords:** Parkinson’s disease, caregiving, relationship satisfaction, communal strength, responsiveness

## Abstract

Parkinson’s disease (PD) is a neurodegenerative disorder which impacts the person’s physical, psychological and relational well-being, and the well-being of their romantic partner, who is often in a caregiving role. People with PD may struggle to empathize with and respond to their partner’s emotional states, which can hinder relationship satisfaction for both partners. Care partners, who may feel burnt out from caring for their partner’s physical and cognitive needs, may be limited in their ability to be responsive to their spouse’s relational needs, which can hinder satisfaction. Despite the challenges faced by couples coping with PD, little work has considered the interpersonal factors associated with relationship satisfaction for affected couples. In the current study, we investigated individual differences in the motivation to be responsive to a partner’s needs (i.e., communal strength), as well as perceptions of a partner’s responsiveness (i.e., the extent to which a person perceives their partner to care for, validate, and understand them). We recruited 20 couples in which one partner was diagnosed with PD and their romantic partner self-identified as a full-time caregiver, in order to examine how responsiveness is associated with both partners’ relationship satisfaction. When partners with PD reported higher communal strength, they reported higher relationship satisfaction and so did their care partner. When partners with PD perceived their care partner to be more responsive, they reported higher relationship satisfaction. These findings provide some preliminary evidence for responsiveness as one interpersonal factor worthy of further consideration for helping couples cope with PD.

## Introduction

Parkinson’s disease (PD) is a multi-dimensional neurodegenerative disorder in which over 800,000 people in North America are diagnosed (Marras et al., 2018). While medications such as L-DOPA, and other forms of therapy can help to reduce symptoms, there is no cure for PD. People with PD (PwPD) are affected by motor symptoms including postural instability, rigidity and tremors (Hoehn & Yahr, 1998), as well as depression, and challenges with facial encoding, empathy, communication, recognizing emotions, and sexual functioning (Bronner et al., 2004; Dujardin et al., 2004; Heiberger et al., 2011; Miller et al., 1996; Narme et al., 2013; Ricciardi et al., 2015). Rates of PD are on the rise, with more than 1,000,000 cases in North America projected for 2030 (Marras et al., 2018). In Canada, 56% of PD patients reported that their romantic partner is their fulltime caregiver (Wong et al., 2014), and these care partners are also negatively affected by their partner’s PD symptoms, which require more support as they progress. Care partners are particularly at risk for care strain when their PwPD is at a more advanced stage of PD (Hand et al., 2019), and report more distress when their partner with PD has greater depression, or cognitive impairments such as reduced empathy (Aarsland et al., 1999; Miller et al., 1996). However, despite the challenges faced by couples coping with PD, there is little dyadic work considering the interpersonal factors associated with both partners’ relationship satisfaction (Mavandadi et al., 2014; Ricciardi et al., 2015). In the current study, we investigated the associations between responsiveness, as well as perceptions of a partner’s responsiveness, and relationship satisfaction for couples coping with PD.

### Well-being in couples coping with PD

Despite the fact that the majority of people with PD are cared for by their romantic partners (Wong et al., 2014) and PD elicits relationship challenges, such as reduced sexual satisfaction for partners with PD and lower emotional awareness, compared to healthy controls (Bronner et al., 2015; Welsh et al., 1997), little work has sought to understand the interpersonal factors associated with relationship satisfaction for both members of these couples. One reason touted for why couples coping with PD report lower relationship satisfaction is because PD symptoms can include impaired empathy (Ricciardi et al., 2015). When PwPD have more empathy impairment, this is associated with lower relationship satisfaction for them, and empathy impairment in people with Alzheimer’s disease has been associated with lower relationship satisfaction for their romantic partners (Martinez et al., 2018; Ricciardi et al., 2015). However, some couples coping with PD remain satisfied despite these challenges. Rusbult et al.’s (1998) Investment Model purports that relationship satisfaction, quality of alternatives, and investment size each influence relationship commitment and the probability of the relationship persisting throughout time. Certainly then, understanding which factors may elicit greater relationship satisfaction for couples coping with Parkinson’s disease could have implications for couples’ relationship commitment and maintenance. Additionally, commitment has been associated with behaviours which promote relationship health such as a desire to accommodate one’s partners’ needs (Rusbult et al., 1998). One interpersonal factor which has been positively associated with the relationship satisfaction in couples coping with PD is *benefit finding:* perceived positive personal growth amidst a challenge (Kim et al., 2007). Benefit finding has been associated with greater relationship quality for both members of couples coping with PD (Mavandadi et al., 2014). Additionally, *mutuality:* reciprocal, positive relationship quality between the caregiver and care-receiver, has been negatively associated with the cognitive and motor challenges of PwPD and caregiver burden, such that when PwPD have more challenges with activities in daily life, or cognition, their care partners report less mutuality, and more burden (Karlstedt et al., 2019). Given this research which points to the importance of understanding a partner’s perspective, and being motivated to maintain a positive, growth mindset to cope with challenges for relationship satisfaction, in the current study, we explore the association between responsiveness and relationship satisfaction for both members of couples in which one partner is coping with PD.

### Responsiveness

A person’s feelings of relationship satisfaction often depend on their own motivation and disposition toward their partner as well as how responsive, supportive, and caring they believe their partner to be toward them (Canevello & Crocker, 2010). In the current study, we consider how one’s motivation to be responsive to their partner’s needs (i.e., their communal strength) as well as their perceptions of their partner’s responsiveness (i.e., perceived partner responsiveness) is associated with both partners’ relationship satisfaction in couples coping with PD.

#### Communal strength

*Communal strength* is the motivation to be responsive to a romantic partner’s needs. In community samples, people higher in communal strength tend to report more intrinsic joy from caring for their partner’s needs, and in turn, both partners report more relationship satisfaction (Le et al., 2018; Lemay et al., 2007; Lemay & Clark, 2008). The majority of the research has documented this association in community samples of couples where there is an expectation of reciprocity of care, but the provision and receipt of communal care has also been shown to maintain satisfaction among couples coping with a health issue. For example, greater communal motivation in caring for a partner with chronic pain has been associated with more relationship satisfaction for both partners (Kindt et al., 2016). For couples coping with a sexual dysfunction, when the partner with the diagnosis is more communal in the specific domain of sexuality, this is associated with greater relationship satisfaction for their romantic partner (Muise et al., 2017). Communal strength may become more salient when one partner is diagnosed with a progressive disorder, such as PD, which may elicit an unbalance in reciprocal care. It is unknown how communal strength may affect the relationship quality of couples when the partner with PD has increasingly more needs which need to be met and is simultaneously less able to meet their romantic partner’s needs. Given that PD limits one’s ability to be empathetic to another’s emotions (Ricciardi et al., 2015), it is possible that the degree of communal strength in PwPD may have important associations with their partner’s relationship satisfaction. Furthermore, how motivated care partners are to respond to their partner’s needs, may also be associated with the relationship satisfaction of the partner with PD.

#### Perceived partner responsiveness

*Perceived partner responsiveness* is the extent to which people believe their partners care for, understand, and validate them (Reis, 2012) and has been associated with more favourable relationship and broader health outcomes. In community samples, when one perceives their partner to be more responsive, which is associated with feeling valued and special, partners report increased sexual desire (Birnbaum et al., 2016), and greater relationship quality and intimacy (Reis et al., 2004). Among clinical samples, perceiving one’s partner to be more responsive is associated with healthier sleep quality for the patient (Selcuk et al., 2017; Slatcher et al., 2015) due to lowered anxiety, as well as faster recovery from health-related incidents (e.g. surgery) (Khan et al., 2009). This is relevant for couples coping with PD because when PwPD perceive their partner to respond to their motor and non-motor challenges well, this may be associated with their relationship satisfaction. Unlike health-related incidents, PD has no cure, and thus it is essential to learn whether perceptions of a partner’s responsiveness can help couples coping with a health-related challenge that is progressive and enduring in nature. Furthermore, if care partners continue to perceive their partner with PD to value them, even despite their challenges, this may help to preserve care partner relationship satisfaction.

## The current study

Despite evidence that both members of couples coping with PD report relational challenges (Mavandadi et al., 2014; Ricciardi et al., 2015) most of the available research has not taken into account both partner’s experience. In other clinical samples, responsiveness has been associated with satisfaction, but participants had a disorder that was physical in nature (e.g. pain). The association between responsiveness and relationship satisfaction has not been tested in clinical samples in which the disorder (e.g. PD) is degenerative and has poignant physical, cognitive and emotional consequences. This is important to investigate because couples coping with PD are at particular risk for relational challenges, as PD may make it more difficult for PwPD to be responsive (Ricciardi et al., 2015) due to emotional awareness challenges, which may negatively impact both the partner with PD, as well as their romantic partner (Martinez et al., 2018). By understanding how responsiveness is associated with the relationship satisfaction for couples coping with PD, this may help to inform effective coping methodologies and interventions for couples where one partner has a progressive neurological disorder. In the current study we build upon previous work indicating the benefits of responsiveness as well as the specific challenges faced by couples coping with PD, to test associations between communal strength and perceived partner responsiveness and relationship satisfaction for PwPD and their care partners. Based on previous work, we expected that when PwPD and care partners reported higher communal strength, and perceived their partner to be more responsive, that they would report higher relationship satisfaction (actor effects) and that one’s score on these predictors would also predict their partner’s relationship satisfaction (partner effects).

## Methods

### Participants and procedures

Couples were recruited from ongoing Dancing with Parkinson’s (DwP) classes from the Greater Toronto Area, and New York City. Couples were eligible to participate if they were in a long-term relationship, in which one partner had PD and the other identified as their primary caregiver. Participants had to be within the ages of 50-90 and able to independently answer survey questions. DwP teachers were contacted via email to verbally promote the study in class, and research assistants also attended classes to recruit participants. Once couples had been individually screened for the eligibility criteria over email, participants were sent unique survey links to complete the online survey on Qualtrics survey platform. Each member of every recruited couple completed every survey measure, and PwPD also completed a measure of PD symptom severity, which care partners did not complete. This protocol was approved by York University’s Ethics Board for the study entitled “Investigating dance on individual and relational outcomes in couples coping with Parkinson’s disease”. Participants were entered to win one of four $25 gift-cards. For this pilot study, we were able to recruit 20 couples.

Participants ranged in age from 62 to 88 (*M* = 73.97, *SD* = 6.9) and had been in their current relationship for 7 to 63 years (*M* = 44.80, *SD* = 13.57). Most participants were married (95%) with 2.5% common-law, and 2.5% living together. All participants were in mixed-sex relationships, in which 9 PwPD were male and 11 were female. PwPD had on average been living with their PD diagnosis for 8.5 years (*SD* = 6.42) and had an average score of 1.24 (*SD* = .67) for non-motor aspects of experiences of daily living, and 1.15 (*SD* = .60) for motor aspects of experiences of daily living, based on the gold-standard MDS-UPDRS questionnaire for PD severity (Goetz et al., 2008) where 0 represents no problems and 4 represents severe challenges. Associations between PD symptom severity and all key variables were considered.

### Measures

#### Communal strength

We assessed communal strength using (Mills et al., 2004) 10-item scale which evaluates a person’s general motivation to be responsive to their partner’s needs. Items were rated on a 10-point Likert scale, from 0 = “not at all” to 10 = “extremely” and included “How happy do you feel when doing something that helps your partner?” α = .68, which compares to reliability in past work, α = .85–.95 (Mills et al., 2004).

#### Perceived partner responsiveness

We assessed how responsive partners perceived their partner to be by using 3 face-valid items from (Reis, 2003) perceived partner responsiveness measure, which assesses how responsive a person feels their partner has been to their needs. Items were rated on a 5-point Likert scale, from 1 = “not at all” to 5 = “very much so”. An example item is “My partner makes me feel cared for”. α= .77, which compares to the reliability in past work with the full scale, α= .97 (Gable et al., 2012).

#### Relationship satisfaction

We assessed relationship satisfaction using two subscales^1^ from the Couples Satisfaction Index (Funk & Rogge, 2007). We used the first 10 items from this 16-item scale. Participants were asked to rate their feelings towards their partner (e.g., extent of happiness) and rate how much they agree with statements such as “our relationship is strong”, scored on a 6-point Likert-scale from 0 = “not true at all” to 5 = “completely true”. α  = .93, which compares to the initial assessment of reliability in previous work using the full scale, α = .98 (Funk & Rogge, 2007).

### Data analysis

Data were restructured to be dyadic by using David Kenny’s data restructuring app, which is available at: https://davidakenny.shinyapps.io/ItoP/. To test our predictions, we used mixed models in SPSS 26, guided by the actor-partner independence model (Kenny et al., 2006). We tested two-level models, with each participant nested within their dyad. Dyads were treated as distinguishable (PwPD and care partner), and thus we had separate intercepts for PwPD and care partners. Each model included both partner’s reports of the predictor variables which were grand mean-centered, and two models were run—one with communal strength as the predictor and one with perceived partner responsiveness as the predictor. The coefficients are unstandardized betas (*b*) and represent changes in the outcome variable for every one-unit change in the predictor from the mean of the sample. To access the data and syntax: https://osf.io/jypqu/?view_only=e5a92873de2648f3b0385062402ff618.

## Results

### Descriptive statistics

Overall, the descriptive statistics for the interpersonal variables indicate that these couples are functioning quite well. In terms of communal strength, partners reported a high degree of motivation to meet their partner’s needs, as both PwPD (*M* = 8.62, *SD* = 1.14) and care partners (*M* = 8.72, *SD* = .921) were past the midpoint of the scale. PwPD did not report less communal strength compared to their care partners, *p* = .762 and when one partner reported being higher in communal strength, this was not significantly associated with their partner being higher in communal strength, see Table 1. Furthermore, when care partners reported more communal strength, their PwPD reported significantly less non-motor challenges (e.g. fatigue, body pain), *r* = -.473.

**Table 1.** Correlations among all key variables and demographics.

Variable	1	2	3	4	5	6	7	8	9	10
1. CP CS	–	.224	.013	.100	.432	–.022	–.473*	.068	.055	.364
2. PwPD CS	–	–	.209	.216	.654**	.555*	–.045	.387	–.054	.576**
3. CP PPR	–	–	–	.545*	.519*	.368	–.114	–.011	–.134	.048
4. PwPD PPR	–	–	–	–	.416	.647**	–.297	–.127	–.077	.119
5. CP Rel. Sat	–	–	–	–	–	.594**	–.230	.086	.019	.312
6. PwPD Rel. Sat.	–	–	–	–	–	–	–.067	.177	.142	.149
7. PD Non-Motor Symptoms	–	–	–	–	–	–	–	.067	–.309	.142
8. PD Motor Symptoms	–	–	–	–	–	–	–	–	.212	.313
9. # Years with PD	–	–	–	–	–	–	–	–	–	–.207
10. Relationship Length	–	–	–	–	–	–	–	–	–	–

*Note*. PwPD = Partner with PD, CP = Care Partner, PPR = Perceived Partner Responsiveness, CS = Communal Strength, Rel. Sat. = Relationship Satisfaction. Relationship length is in years. *p <.05, **p < .01.

Additionally, PwPD perceived their partners to be quite responsive (*M* = 4.55, *SD* = .54) as did care partners (*M* = 4.61, *SD* = .39) as indicated by these means being at the upper ends of the scale. PwPD were not perceived as significantly less responsive to their care partner’s needs, compared to how responsive they perceived their care partners to be, *p* = .660, and when a PwPD reported perceiving their partner as more responsive, their care partner reported significantly higher perceived partner responsiveness, *r* = .545. However, when either partner reported higher communal strength, this was not significantly correlated with their partner perceiving them as more responsive. In terms of outcome variables, relationship satisfaction was relatively high on average, for PwPD (*M* = 40.70, *SD* = 8.30) and care partners (*M* = 42.05, *SD* = 5.76) out of a total of 51 with higher scores being indicative of higher satisfaction. PwPD were not significantly less satisfied in their relationship, compared to their care partners *p* = .556. Importantly, PD symptom severity was quite low in this sample, which is perhaps why we did not see any significant differences in our key variables between PwPD and their care partners.

### Tests of our key predictions

#### Communal strength

**Figure 1.**
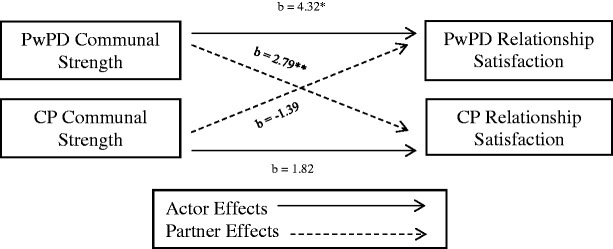
Actor and partner effects of communal strength on relationship satisfaction. *Note*. * = *p* <.05. PwPD = partner with PD, CP = care partner.

When PwPD reported higher communal strength, they reported significantly higher relationship satisfaction, and so did their care partner, see Table 2 and Figure 1. Care partners’ self-reported communal strength was not related to either partners’ relationship satisfaction. These findings indicate that in this sample, the self-reported CS of the PwPD is linked to both partner’s relationship satisfaction.

**Table 2.** Effects of communal strength and perceived partner responsiveness on relationship satisfaction.

	PwPD’sRelationship satisfaction			CP’sRelationship satisfaction		
	*b* (SE)	*t*	CI	*b* (SE)	*t*	CI
PwPD CS	4.32 (1.49)	25.24*	1.17, 7.48	2.97 (.88)	3.37**	1.11, 4.83
CP CS	–1.39 (1.84)	–.756	–5.30, 2.50	1.82 (1.09)	1.67	–.480, 4.12
PwPD PPR	9.78 (3.39)	2.89*	2.63, 16.93	2.01 (2.57)	.783	–3.41, 7.44
CP PPR	.455 (4.67)	.097	–9.41, 10.32	6.07 (3.55)	1.71	–1.42, 13.56

*Note. b* represents unstandardized betas; SE represents standard error of the estimate. PwPD = partner with PD (N = 20), CP = care partners (N = 20). *p <.05. **p <.01

#### Partner perceived responsiveness

**Figure 2.**
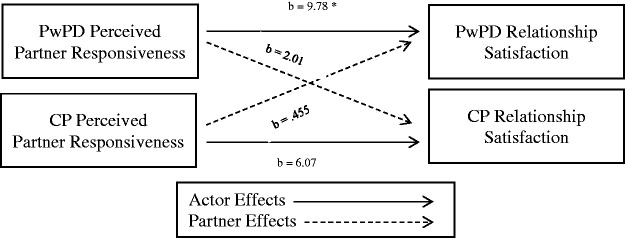
Actor and partner effects of perceived partner responsiveness on relationship satisfaction. *Note*. * = *p* <.05. PwPD = partner with PD, CP = care partner.

As predicted, when PwPD perceived their partner to be more responsive, they reported significantly higher relationship satisfaction, see Table 2 and Figure 2. There were no significant partner effects of perceived partner responsiveness.

### Additional analyses

Age, sex, and PD symptom severity and duration were not significantly correlated with any of our key variables. However, when relationships were longer relative to shorter, PwPD reported significantly higher communal strength. Therefore, we tested whether effects of communal strength on relationship satisfaction remained significant, after relationship length was controlled. After accounting for relationship length, all of the effects reported above remained significant.

## Discussion

Maintaining relationship satisfaction for couples coping with progressive disorders is challenging, as PwPD have accelerating symptoms, and care partners may experience burden (Caap-Ahlgren & Dehlin, 2002). Despite these difficulties, we found that in a sample of couples which have been in their relationship for more than 40 years on average, and without severe PD symptoms, both partners can demonstrate responsiveness and report high relationship satisfaction. In the current study, PwPD who are higher in communal strength (i.e., more motivated to meet their partner’s needs) report higher relationship satisfaction and so does their care partner. In addition, when PwPD perceived their care partner to be more responsive (i.e., felt cared for their partner), they were also more satisfied.

### Contribution to research on responsiveness

This work contributes to a growing body of research in both community and clinical samples on the benefits of communal strength and perceived partner responsiveness for relationship satisfaction. Previous work on couples coping with a pain related disorder (i.e., pain during sex) found that when the affected partner (in this case the woman) was higher in communal strength, specifically in the domain of sexuality, her partner reported higher relationship satisfaction (Muise et al., 2017). In other research, a spouse providing care for a partner with chronic pain who is more, compared to less, communal reported feeling more satisfied in the relationship (Kindt et al., 2016). The current findings echo these results and extend them to a sample of couples coping with a physical, cognitive, emotional disorder which is degenerative in nature. That is, when the partner with PD reported higher communal strength, both partners reported higher satisfaction. However, it is important to note that in a sample with more severe PD symptoms, these results may have differed, based on previous work in which severe empathy impairment in PwPD has been associated with lower relationship satisfaction for them, and severe empathy impairment in people living with Alzheimer’s disease has been associated with lower relationship satisfaction for their care partners (Martinez et al., 2018; Ricciardi et al., 2015).

Additionally, in our study there was no association between the care partner’s communal strength and either partner’s relationship satisfaction. Perhaps this could be because care partners are generally expected to be motivated to meet their partner’s needs (Kroemeke et al., 2019), but when the partner with PD remains motivated to respond to their partner despite coping with PD, this reinforces the mutual effort to care for one another, despite different roles within the relationship. It is also possible that care partners may not experience the benefits of being communal if they are caring for their partner to such an extent that they neglect their personal needs (unmitigated communion) (Le et al., 2018). While our study did not consider unmitigated communion, it could be investigated in future work, as higher degrees of unmitigated communion in care partners has been associated with distress, and poorer patient adjustment among couples coping with health issues such as breast cancer, and a coronary event (Helgeson, 1993, 2003). Moreover, our study did not find a significant association between higher communal strength and being perceived as more responsive by one’s partner. While this may be due to a lack of power, it is also possible that not all forms of support or communal care are always perceived by one’s partner. *Invisible support* which is provided, but not directly perceived by the recipient (Bolger et al., 2000) has been associated with relational benefits, at times even more so than perceivable support (Howland & Simpson, 2010). In the context of dyads coping with neurodegenerative disorders, the nuances of perceived or invisible support may be fruitful to consider in future research.

While work in community samples has investigated associations between perceived partner responsiveness and relationship satisfaction (Reis et al., 2004), to our knowledge, our study is the first to consider how perceived partner responsiveness may affect relational, rather than health-related outcomes, in a sample of couples coping with a progressive condition. Our descriptive analyses seem to suggest that when care partners report higher communal strength, this was associated with fewer non-motor challenges for their PwPD (e.g. body pain) which appears to be consistent with past work on the physical benefits of responsive care (Khan et al., 2009; Selcuk et al., 2017). A key extension of the current work is that we considered how perceptions of partner responsiveness were related to broader relational outcomes in sample of couples coping with a degenerative disease. When the partner with PD perceived their care partner to be more responsive, they were more satisfied in their relationship. Previous work suggests that PwPD may struggle to empathize with others (Ricciardi et al., 2015). While we did not assess empathy directly, we did find that PwPD were perceived to be highly responsive by their romantic partners, perhaps due to the low PD symptom severity in the current sample.

### Implications for couples coping with PD and program development

The findings from the current study add to what is known about the dyadic nature of coping with PD, with regards to how responsiveness is associated with relationship satisfaction. Given that in our sample, the degree of communal strength of the partner with PD was associated with care partner relationship satisfaction, this indicates that responsiveness might be a promising factor to explore for maintaining care partner well-being.

The findings of the current pilot study are a first step in the theoretical development of new programs aimed at couples coping with PD. For example, if an intervention could be created which aims to increase responsiveness, subsequent increases in relationship satisfaction could be tested. It will be important to distinguish if such interventions which emphasize responsiveness are efficient only for couples where PD symptoms are less severe, as in our sample, compared to couples where the PwPD experiences more severe symptoms. Future programming for these couples could make use of creative activities in which partners interact and show responsiveness in salient ways such as partnered dancing or other forms of artistic collaboration. Correspondingly, dancing for couples coping with PD has been linked to improved state of mind, and quality of life for both PwPD and care partners (Heiberger et al., 2011), but mechanisms which may help to explain these positive outcomes (e.g., perceived partner responsiveness) have not yet been empirically investigated. Arts-based activities can be inclusive and enjoyable for people living with a variety of abilities. For example, Dance for PD ® can be fully carried out in seated position, with minimal gross-motor movements (DanceforPD, 2017). Additionally, in response to COVID-19, many recreational activities for PwPD and their care partners have transitioned to virtual formats on platforms such as Zoom, such as Dancing with Parkinson’s Canada (Hay, 2020) which may be more inclusive for PwPD with limited access to other forms of recreation or leisure to participate in with their care partner at this time.

### Limitations and future directions

Limitations of the present study include the sample size, the uniqueness of the sample, and the cross-sectional nature of the data. Given the pilot-nature of the present study, our ability to be confident in our dyadic results is hindered, based on relatively low sample size. Furthermore, because participants were well enough to attend a dance class, it is quite possible that this restricted the range of PD symptom severity in our sample, which affects the generalizability of our results. Nonetheless, our sample was able to obtain a balance of male and female care partners, and our sample size is on par with other dyadic studies investigating couples coping with neurological disorders (e.g., Martinez et al., 2018; Mavandadi et al., 2014). Given that the study design was a one-time survey, while this gives us a snapshot of how couples are coping with PD, we cannot account for any changes in relationship satisfaction over time. It would be beneficial for future research to consider the longitudinal role of responsiveness on relationship satisfaction among couples coping with PD; specifically, if these associations are replicated in samples with more severe symptoms, as PD progresses.

Despite these caveats, this work serves as a preliminary step to considering how couples cope with PD together, identifying associations between communal strength, perceived partner responsiveness and relationship satisfaction in a clinical sample of couples coping with a degenerative disorder. The findings of this work may help to encourage more dyadic work in this population, by showing that online research with couples coping with PD is possible, particularly when symptom severity is not high. At this present time, research on interventions which target couples coping with PD are important as COVID-19 heightens the risk for aging persons to feel isolated, and distressed (Chu et al., 2020).

It is essential that future work on couples’ coping with neurological disorders continues to take into account partner effects. While the majority of research on care partners only considers their participation and responses (e.g. Caap-Ahlgren & Dehlin, 2002; Miller et al., 1996; Schrag et al., 2006), our study, as well as other pilot studies (e.g. Mavandadi et al., 2014), have emphasized the importance of taking into account how both partner’s experiences and qualities predict their partner’s personal and relational well-being. While more dyadic work should be conducted with larger sample sizes, this will likely not be without challenges. In the present study, it was difficult to find couples where PwPD did not have mobility or cognitive challenges which prevented them from answering the online survey independently (e.g. a tremor which hindered filling out an online survey). This problem greatly compromised the sample size, as well as the variability of PD symptom severity range in our sample. Due to the fact that the survey was relational in nature, we could not have care partners assist their partner with PD, as this would introduce too much bias in the responses. Perhaps this is why there is scant amounts of dyadic research on couples coping with neurodegenerative diseases. In order to yield larger sample sizes, future researchers could obtain data from PwPD through in-person or virtual interviews, in which the researcher administers the measures, and takes note of their responses. Though this would be more time-consuming and costly, it would enable more meaningful dyadic data to be acquired for this population of couples, which will be growing in the forthcoming decades.

## Conclusion

The current pilot study investigated the benefits of communal strength and perceived partner responsiveness on relationship satisfaction for couples coping with PD. The results demonstrated associations between PwPD’s communal strength on their own, as well as their care partner’s relationship satisfaction, and when PwPD perceive their romantic partner as more responsive, they are more satisfied in their relationship. These findings identify responsiveness as factor worthy of further exploration in this population. Our hope is that this initial work inspires future dyadic research on couples coping with PD that can assess both partners’ relational, psychological and physical well-being over time.

## Supplemental Material

sj-pdf-1-prx-10.1177_0033294121998032 - Supplemental material for Responsiveness and Relationship Satisfaction in Couples Coping With Parkinson’s Disease: A Pilot Study Click here for additional data file. Supplemental material, sj-pdf-1-prx-10.1177_0033294121998032 for Responsiveness and Relationship Satisfaction in Couples Coping With Parkinson’s Disease: A Pilot Study by Eden Rose Champagne and Amy Muise in Psychological Reports

## Note

1 The final subscale (6-items) was not included due to an error in programming
