# Supplementary material for: Responsiveness and Relationship Satisfaction in Couples Coping With Parkinson’s Disease: A Pilot Study
Source: Psychol Rep. 2021 Feb 24;125(2):804–21. doi: 10.1177/0033294121998032 (PMC9003747; doi:10.1177/0033294121998032)
Supplement: sj-pdf-1-prx-10.1177_0033294121998032 - Supplemental material for Responsiveness and Relationship Satisfaction in Couples Coping With Parkinson’s Disease: A Pilot Study [file sj-pdf-1-prx-10.1177_0033294121998032.pdf]

Table 1

Correlations Among all Key Variables and Demographics

Variable	1	2	3	4	5	6	7	8	9	10
1. CP CS	-	.224	.013	.100	.432	-.022	-.473*	.068	.055	.364
2. PwPD CS	-	-	.209	.216	.654**	.555*	-.045	.387	-.054	.576**
3. CP PPR	-	-	-	.545*	.519*	.368	-.114	-.011	-.134	.048
4. PwPD PPR	-	-	-	-	.416	.647**	-.297	-.127	-.077	.119
5. CP Rel. Sat	-	-	-	-	-	.594**	-.230	.086	.019	.312
6. PwPD Rel. Sat.	-	-	-	-	-	-	-.067	.177	.142	.149
7. PD Non-Motor Symptoms	-	-	-	-	-	-	-	.067	-.309	.142
8. PD Motor Symptoms	-	-	-	-	-	-	-	-	.212	.313
9. # Years with PD	-	-	-	-	-	-	-	-	-	-.207
10. Relationship Length	-	-	-	-	-	-	-	-	-	-

Note. "PwPD" = Partner with PD, "CP" = Care Partner, "PPR" = Perceived Partner Responsiveness, "CS" = Communal Strength, "Rel. Sat". = Relationship Satisfaction. Relationship length is in years. * $p < .05$, ** $p < .01$.

Table 2

Effects of Communal Strength and Perceived Partner Responsiveness on Relationship Satisfaction

	PwPD's Relationship Satisfaction			CP's Relationship Satisfaction		
	b (SE)	*t*	CI	*b* (SE)	*t*	CI
PwPD CS	4.32(1.49)	25.24*	1.17, 7.48	2.97(.88)	3.37**	1.11, 4.83
CP CS	-1.39(1.84)	-.756	-5.30, 2.50	1.82(1.09)	1.67	-.480, 4.12
PwPD PPR	9.78(3.39)	2.89*	2.63, 16.93	2.01(2.57)	.783	-3.41, 7.44
CP PPR	.455(4.67)	.097	-9.41, 10.32	6.07(3.55)	1.71	-1.42, 13.56

Note. b represents unstandardized betas; SE represents standard error of the estimate. PwPD = partner with PD (N = 20), CP = care partners (N = 20). * $p < .05$. ** $p < .01$

Figure 1

Actor and Partner Effects of Communal Strength on Relationship Satisfaction

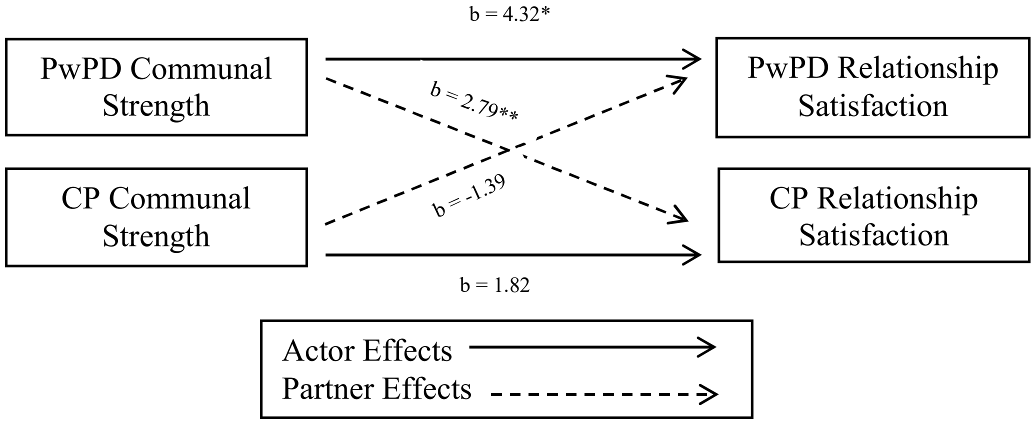

Note. * = p <.05. PwPD = partner with PD, CP= care partner.

Figure 2

Actor and Partner Effects of Perceived Partner Responsiveness on Relationship Satisfaction

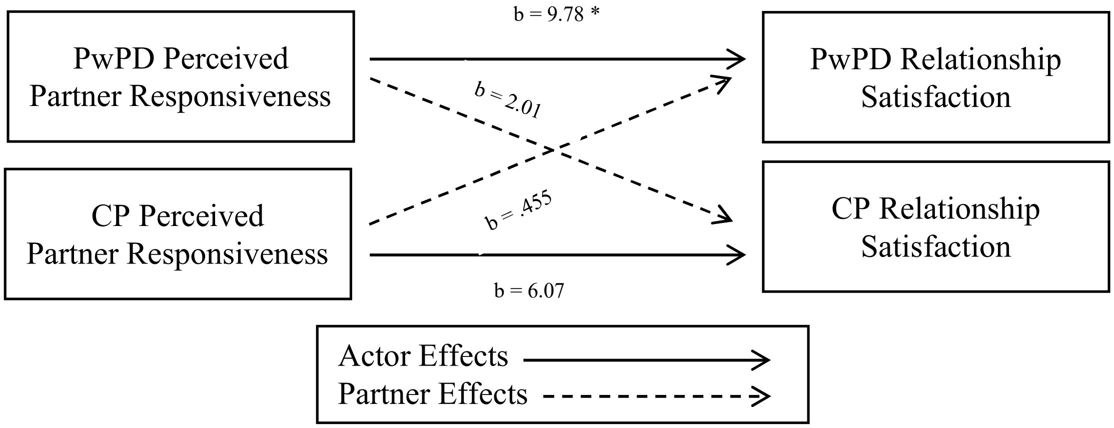

Note. * = p <.05. PwPD = partner with PD, CP= care partner.
